# The Origins of ST11 KL64 Klebsiella pneumoniae: a Genome-Based Study

**DOI:** 10.1128/spectrum.04165-22

**Published:** 2023-03-27

**Authors:** Junna Wang, Yu Feng, Zhiyong Zong

**Affiliations:** a Center of Infectious Diseases, West China Hospital, Sichuan University, Chengdu, China; b Center for Pathogen Research, West China Hospital, Sichuan University, Chengdu, China; c Division of Infectious Diseases, State Key Laboratory of Biotherapy, Chengdu, China; Department of Clinical Laboratory, Peking University People's Hospital, Beijing, China

**Keywords:** carbapenem resistance, KPC, carbapenemase, *Klebsiella pneumoniae*, *Klebsiella*

## Abstract

Carbapenem-resistant Klebsiella pneumoniae (CRKP) is a major severe threat for human health, and its spread is largely driven by a few dominant lineages defined by sequence types (ST) and capsular (KL) types. ST11-KL64 is one such dominant lineage that is particularly common in China but also has a worldwide distribution. However, the population structure and origin of ST11-KL64 K. pneumoniae remain to be determined. We retrieved all K. pneumoniae genomes (*n* = 13,625, as of June 2022) from NCBI, comprising 730 ST11-KL64 strains. Phylogenomic analysis of core-genome single-nucleotide polymorphisms identified two major clades (I and II) plus an additional singleton of ST11-KL64. We performed dated ancestral reconstruction analysis using BactDating and found that clade I likely emerged in 1989 in Brazil, while clade II emerged around 2008 in eastern China. We then investigated the origin of the two clades and the singleton using a phylogenomic approach combined with analysis of potential recombination regions. We found that ST11-KL64 clade I is likely a hybrid with 91.2% (ca. 4.98 Mb) of the chromosome derived from the ST11-KL15 lineage and 8.8% (483 kb) acquired from ST147-KL64. In contrast, ST11-KL64 clade II was derived from ST11-KL47 with swapping of a 157-kb region (3% of the chromosome) containing the capsule gene cluster with clonal complex 1764 (CC1764)-KL64. The singleton also evolved from ST11-KL47 but with swapping of a 126-kb region with ST11-KL64 clade I. In conclusion, ST11-KL64 is a heterogenous lineage comprising two major clades and a singleton with different origins that emerged in different countries at different time points.

**IMPORTANCE** Carbapenem-resistant Klebsiella pneumoniae (CRKP) has emerged as a severe threat globally and is associated with increased lengths of hospital stay and high mortality in affected patients. The spread of CRKP is largely driven by a few dominant lineages, including ST11-KL64, the dominant type in China with a worldwide distribution. Here, we tested the hypothesis that ST11-KL64 K. pneumoniae is a single genomic lineage by performing a genome-based study. However, we found that ST11-KL64 comprises a singleton and two major clades, which emerged in different countries in different years. In particular, the two clades and the singleton have different origins and acquired the KL64 capsule gene cluster from various sources. Our study underscores that the chromosomal region containing the capsule gene cluster is a hot spot of recombination in K. pneumoniae. This represents a major evolutionary mechanism employed by some bacteria for rapid evolution with novel clades that accommodate stress for survival.

## INTRODUCTION

Klebsiella pneumoniae is a major opportunistic pathogen that causes both community- and hospital-acquired infections worldwide (1, 2). In particular, carbapenem-resistant K. pneumoniae (CRKP) is associated with prolonged lengths of hospital stay and increased mortality for infected patients, and its emergence represents a severe threat for human health globally (3). The clinical prevalence of CRKP has been largely attributed to severe dominant sequence types (ST), particularly ST258 and ST11, both belonging to the clonal group (CG) 258 (4–8). ST258 is disseminated worldwide and has been proposed to be a hybrid in which 80% of its genome derived from that of ST11 and 20% from ST442 (5). ST11 is particularly common in Asia and South America (8, 9). ST11 can be further assigned to several capsular types (KL), including KL15, KL47, and KL64, among which ST11-KL64 is the dominant CRKP type in China (10, 11). Study of the origin and population structure of certain bacterial clones may provide much-needed insights into understanding why such clones become successful and may help to design targeted countermeasures. However, the origin and the population structure of ST11-KL64 remain to be determined. To address this, we took advantage of a large number of K. pneumoniae sequences isolates available in the National Center for Biotechnology Information (NCBI; https://www.ncbi.nlm.nih.gov) and performed a detailed genome-based analysis.

We identified that ST11-KL64 is a heterogenous lineage comprising two major clades and a singleton with different origins, different sources of the KL64 capsule gene cluster, and different places and time points of emergence. Our findings led to the identification of unique genetic traits of ST11-KL64 and may enhance the understanding toward the success of this clinically important CRKP lineage.

## RESULTS

### There were 730 ST11-KL64 strains with genome sequences available, seen in East Asia, South America, and Europe.

**FIG 1:**
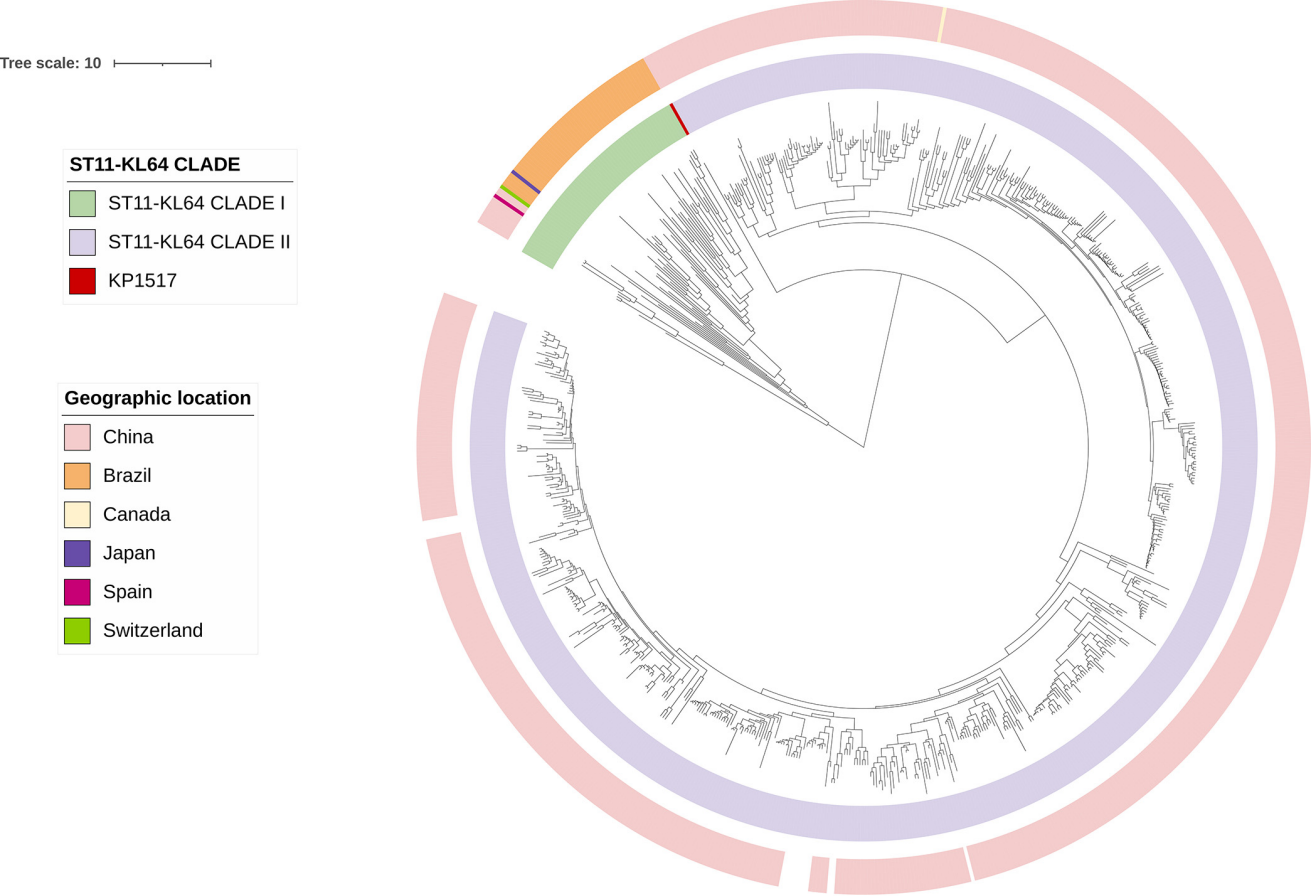
The phylogenomic tree of 730 ST11-KL64 K. pneumoniae strains. The tree was inferred using strain 090357 (accession number CP066523) as the reference. The phylogeny was inferred from core SNPs under a GTR model with site rate variation and a 100-bootstrap test. The outer circle is the geographic location, and the inner circle exhibits the two clades and the singleton (KP1517). The scale bar represents the number of nucleotide substitutions per site.

There were 13,625 K. pneumoniae genome assemblies in NCBI as of 1 June 2022 (see Data Set S1 in the supplemental material). As quality control, 1,039 genomes were discarded from further analysis due to duplicated biosamples (*n* = 103), low-quality assembly as defined by NCBI (e.g., excessive frameshifted proteins and fragmented assembly; *n* = 674), completeness of <95% (*n* = 51), contamination of >5% (*n* = 25), heterogeneity of >50% (*n* = 162), or genomes belonging to species other than K. pneumoniae (*n* = 24). The remaining 12,586 genomes (Data Set S2) were included for further study and comprised 730 ST11-KL64 genomes (Data Set S3). Among 730 ST11-KL64 strains, the geographical location was available for 713 with most (*n* = 659; 659/730, 90.3%) from China, 50 (6.8%) from Brazil, and one from each of Spain, Switzerland, Canada, and Japan (Fig. 1). Among ST11-KL64 strains for which the year of recovery was available, the earliest one was recovered in 2006 from Brazil (Data Set S3). The vast majority (*n* = 704, 96.4%) of the 730 ST11-KL64 strains had genes encoding known carbapenemases, including KPC-2 in 686 (94.0%, 686/730), NDM-1 in 9 (1.2%), OXA-48 in 10 (1.4%), NMD-5 in 4 (0.5%), and KPC-12, KPC-30, and KPC-41 in 2 strains each. Among these strains, 12 had genes encoding KPC-2 plus another carbapenemase, either NDM-1 (in 7 strains), NDM-5 (in 4), or OXA-48 (in 1).

### ST11-KL64 comprised two major clades, which emerged at different time points.

Phylogenomic analysis of 730 ST11-KL64 strains based on core-genome single-nucleotide polymorphisms (SNPs) after removing recombination clearly illustrated the presence of two highly supported distinct major clades of ST11-KL64, here referred to as clade I (comprising 64 strains) and clade II (the remaining strains) (Fig. 1). Of note, a single strain, KP1517, was well separated from all other strains of clade II by a long branch (Fig. 1). This suggested that KP1517, which was recovered in 2018 in China, is likely a singleton and may have a different origin from all other ST11-KL64 strains. We therefore excluded KP1517 from clade II and regarded it as a singleton in further analyses. Clade I comprises strains from Brazil, China, Japan, Spain, and Switzerland. In contrast, all but one clade II strains with geographical information available were from China, and the remaining one was from Canada (Data Set S3).

**FIG 2:**
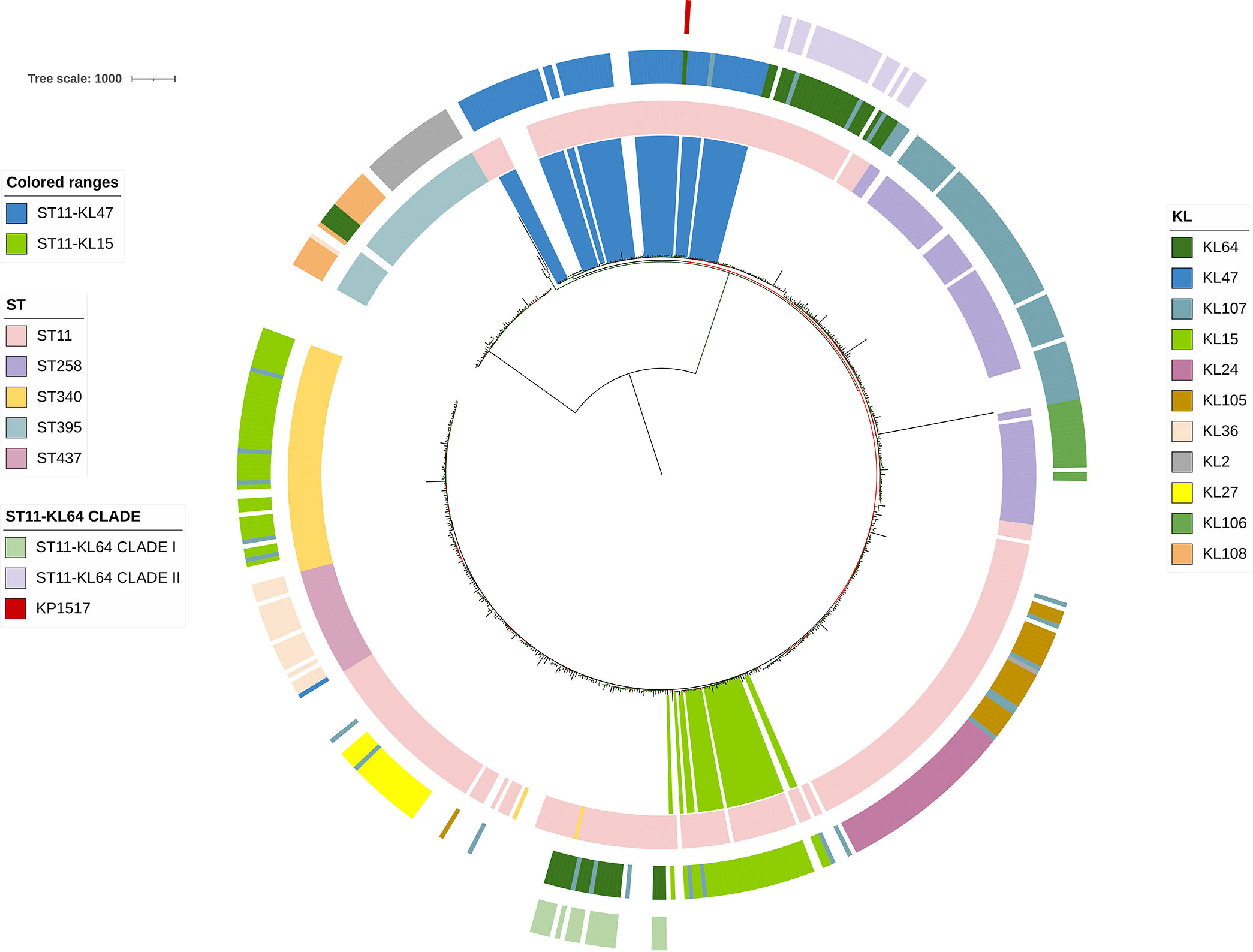
The phylogenomic tree of 565 K. pneumoniae strains belonging to the branch containing ST11-KL64. The tree was inferred using strain 090357 (accession number CP066523) as the reference. The phylogeny was inferred from core SNPs under a GTR model with site rate variation and a 100-bootstrap test. The tree was midpoint-rooted with bootstrap support over 50% shown in gradients. The circles from the inner to the outer represent sequence types (ST), KL (capsular) types, and ST11-KL64 clades, respectively. The colored ranges represent ST11-KL47 and ST11-KL15 strains. The scale bar represents the number of nucleotide substitutions per site.

To elucidate the origin of the two major clades of ST11-KL64, we first performed dereplicating using dereplicator.py (minimum pairwise distance, 0.001) and obtained 4,826 more unique K. pneumoniae genomes (Data Set S4), including 43 ST11-KL64 that remained (Data Set S5) from the included 12,586 genomes. We then performed a recombination-free phylogenomic analysis by comparing the 4,826 more-unique K. pneumoniae genomes. There were 565 genomes (Data Set S6) belonging to the branch containing all ST11-KL64 ones in the phylogenomic tree inferred by the first iteration of Gubbins (Fig. S1) and these were further selected for completing all five iterations of Gubbins to infer a fine-scale phylogenomic analysis after removing recombination regions (Fig. 2). In contrast, KL64 strains belonged to 37 STs (Data Set S7) and were distributed across the phylogenomic tree based on the 4,826 K. pneumoniae genomes (Fig. S1). The phylogenomic tree identified that ST11-KL15 and ST11-KL47 were the sister lineage of clade I and clade II, respectively. This suggested that the two ST11-KL64 clades likely have different origins.

**FIG 3:**
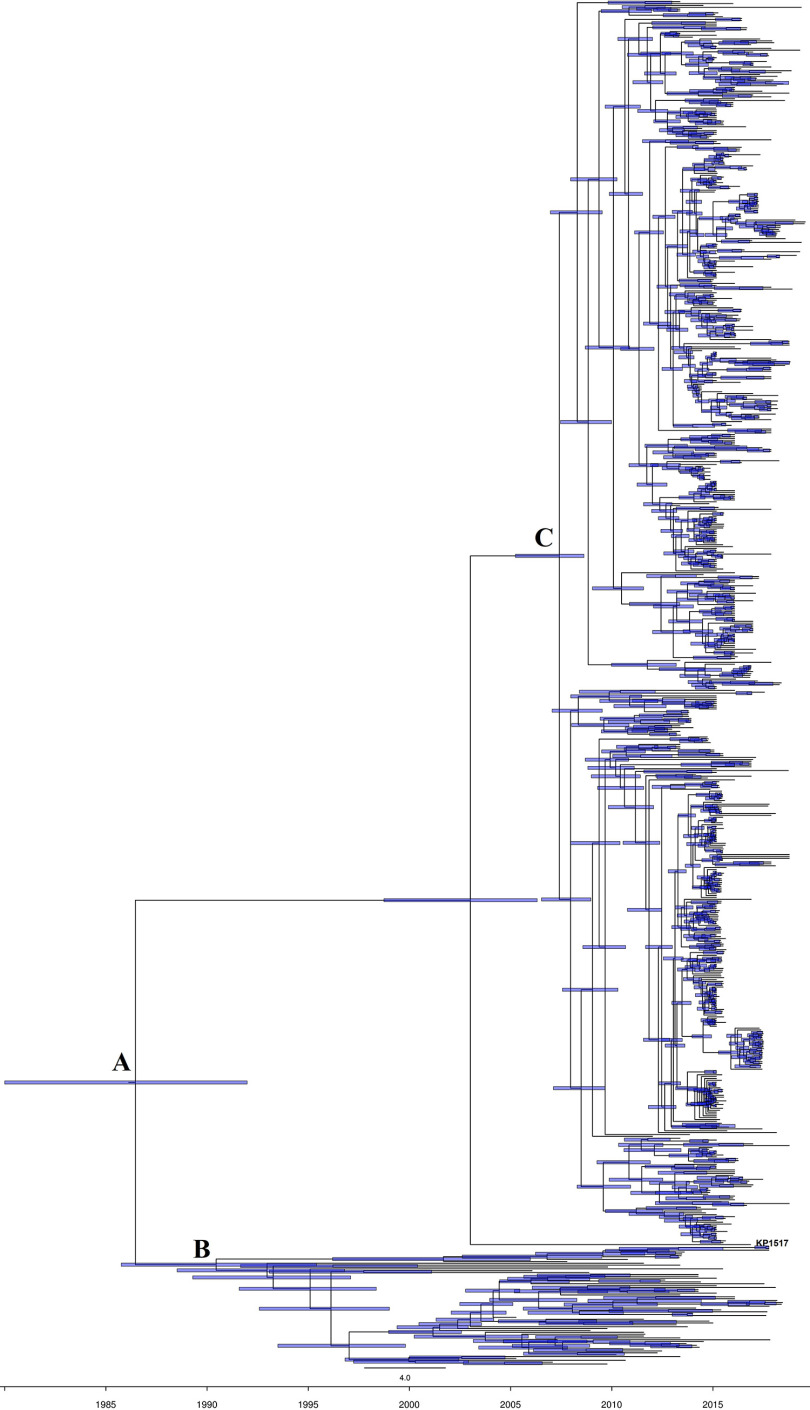
The dated phylogenomic tree of 730 ST11-KL64 strains. The tree was inferred using BactDating v1.0.1 after correction for recombination using Gubbins v3.1.6. Point A, the MRCA of the ST11-KL64 lineage, was estimated to have emerged in January 1985 (95% CI, November 1977 to February 1991). Point B, the MRCA of ST11-KL64 clade I, was dated to June 1989 (95% CI, March 1984 to January 1995). Point C, the MRCA of ST11-KL64 clade II, was dated to April 2008 (95% CI, November 2005 to August 2009).

We performed a molecular clock analysis using BactDating v1.0.1 (12) to date the emergence of ST11-KL64 and its two clades (Fig. 3). The refined analysis revealed an average point mutation rate μ of 5.55 (95% confidence interval [CI], 5.00 to 6.18) substitutions per year for ST11-KL64 strains and a root date of January 1985 (95% CI, November 1977 to February 1991) (Fig. 3). The most recent common ancestor (MRCA) of ST11-KL64 clade I and clade II was dated to June 1989 (95% CI, March 1984 to January 1995) (Fig. 3) and April 2008 (95% CI, November 2005 to August 2009) (Fig. 3), respectively. By aligning their geographical locations, it became evident that the two major clades emerged at different time points in different countries. For clade I, the earliest strain was recovered in 2006 in Brazil, suggesting that this country was likely the place of the emergence of clade I. For clade II, the earliest strains were reported in Zhejiang and Jiangsu Provinces of China, both of which are located in the Yangtze Delta region (Data Set S3). This highlighted that clade II likely emerged in this eastern China region and thereafter spread across China.

### ST11-KL64 clade I derived from ST11-KL15 and acquired a 483-kb large region containing the capsule gene cluster from ST147-KL64 by recombination.

We next investigated the origin of clade I. The phylogenomic analysis based on the 565 genomes described above indicated that ST11-KL15 was a sister lineage of ST11-KL64 clade I (Fig. 2). Strains of ST11-KL64 clade I had 1,577 SNPs (median; range, 1,210 to 2,277) with ST11-KL15 strains (*n* = 29). Most (1,438) of the SNPs concentrated in a 483-kb chromosomal region, which accounted for 8.8% of the complete chromosome corresponding to nucleotide positions 1,671,090 to 2,153,913 of the chromosome of reference strain 090357 (accession number CP066523), containing the capsular gene cluster (Fig. S2). The concentration of these SNPs suggested a foreign origin of the 483-kb large region, which was likely due to recombination. The MRCA of ST11-KL15 was dated to February 1984 (95% CI, May 1973 to July 1992) (Fig. S3), earlier than the emergence of ST11-KL64 clade I. This supported that ST11-KL64 clade I derived from ST11-KL15 rather than vice versa.

**FIG 4:**
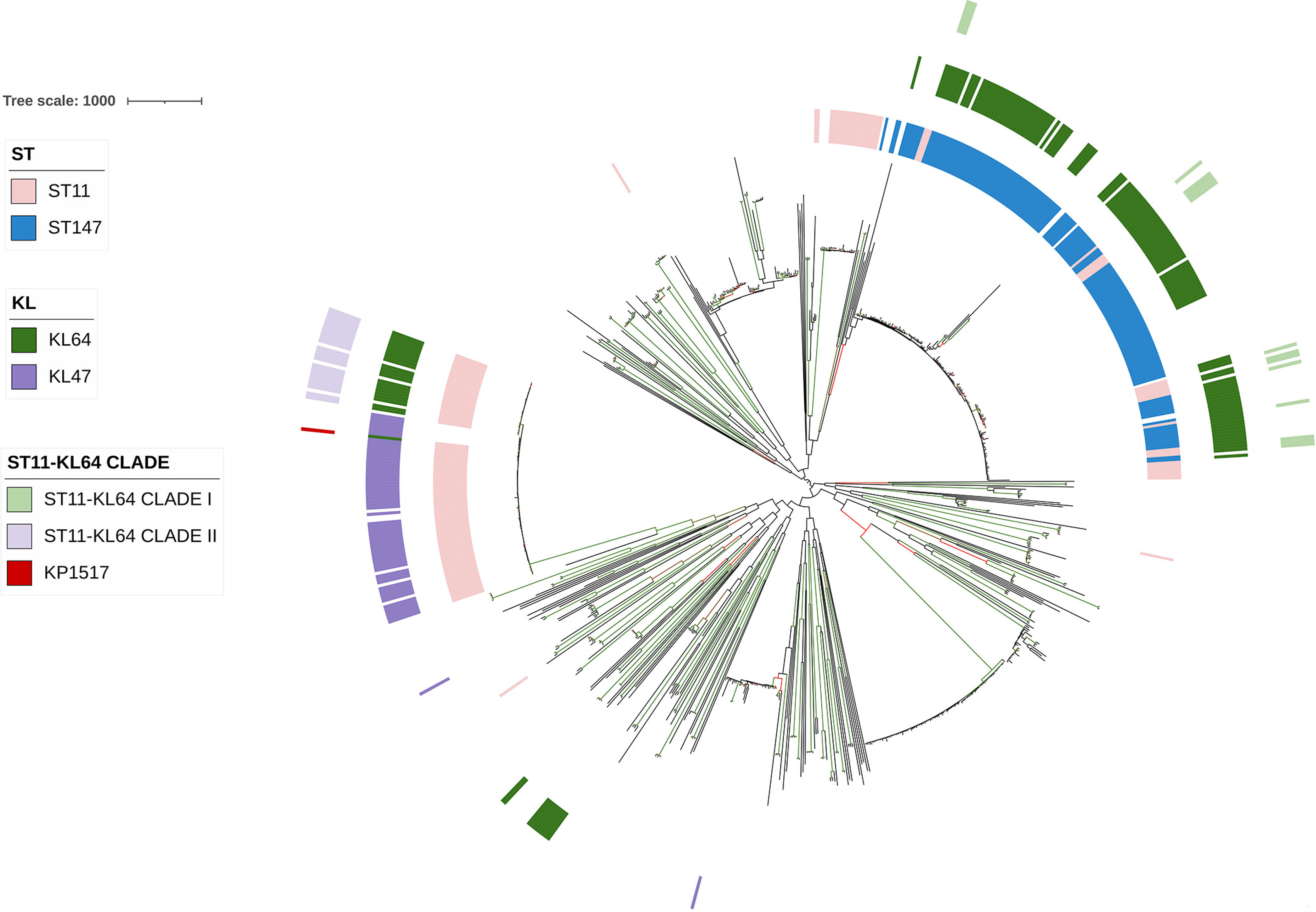
The phylogenetic tree of 857 K. pneumoniae strains based on the 483-kb recombination region. The tree was inferred using strain 090357 (accession number CP066523) as the reference. The phylogeny was inferred from core SNPs under a GTR model with site rate variation and a 100-bootstrap test. The tree was midpoint-rooted with bootstrap support of over 50% shown in gradients. The circles from the outer to the inner represent ST11-KL64 clades, KL64 or KL47 strains (regardless of STs), and ST11 or ST147 strains, respectively. The scale bar represents the number of nucleotide substitutions per site.

**FIG 5:**
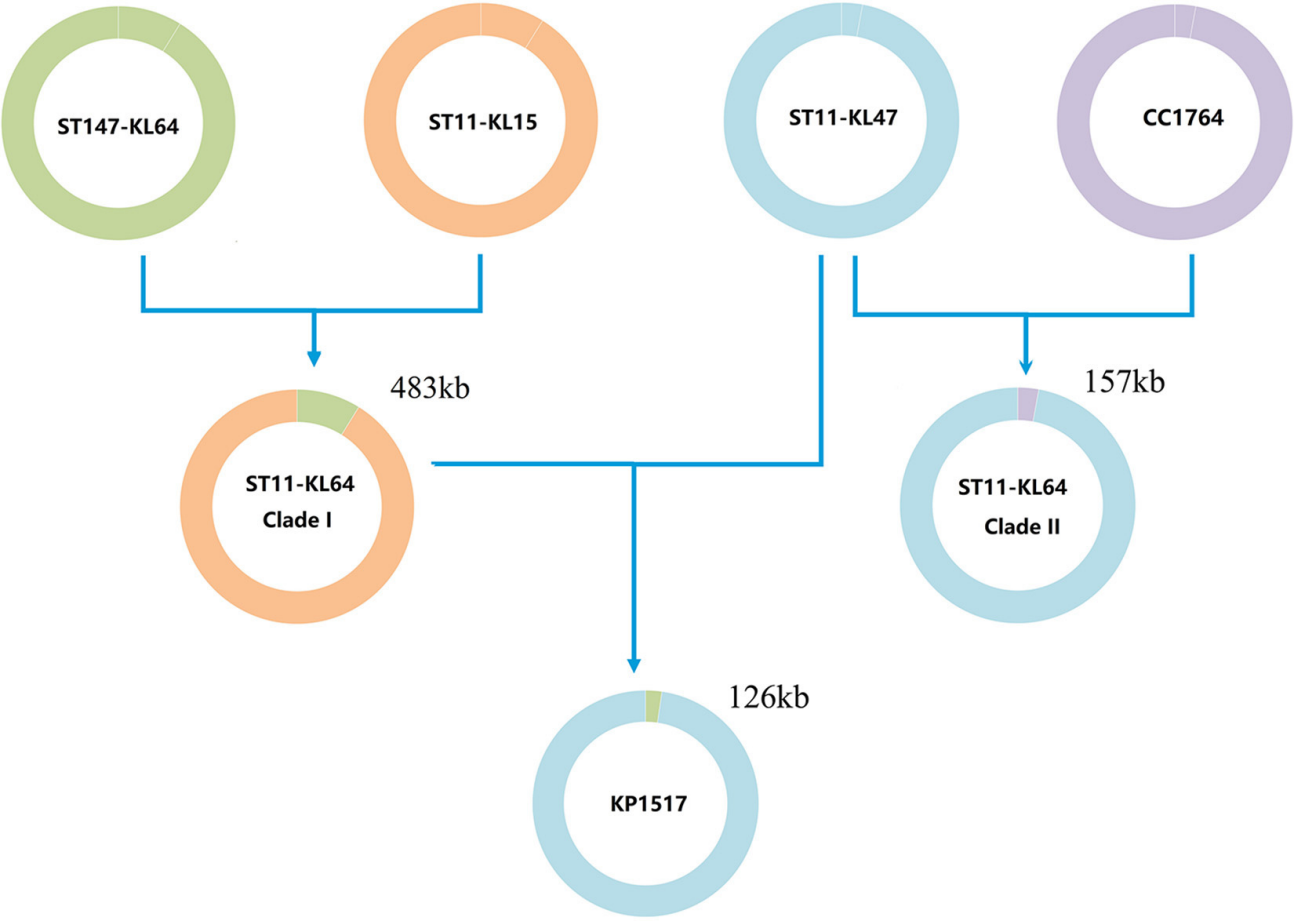
Schemes for the formation of ST11-KL64 clade I, clade II, and the singleton KP1517. Main nodes of interest are shown to demonstrate the different recombination events leading to the formation of the two clades and the singleton.

To identify the possible donor of the recombination region, we performed a phylogenetic analysis of the 4,826 more-unique K. pneumoniae genomes for the 483-kb region. The first iteration of Gubbins identified 857 genomes belonging to the branch containing all ST11-KL64 ones in the phylogenetic tree based on the alignment of this region (Fig. S4). The 857 genomes were further selected for completing all five iterations of Gubbins to infer a fine-scale phylogenetic tree (Fig. 4). ST147-KL64 was identified as the sister lineage of the ST11-KL64 clade I for this 483-kb region. The MRCA of the ST147-KL64 lineage was dated to May 1894 (95% CI, November 1866 to July 1921) (Fig. S5), much earlier than the emergence of ST11-KL64 clade I. Among the 857 genomes, strains of ST11-KL64 clade I had 20,691 SNPs (median; range, 20,602 to 21,914) with ST147-KL64 strains (*n* = 103), suggesting a remote evolutionary distance (Fig. S2). However, the 483-kb region was nearly identical between strains of ST11-KL64 clade I and those of ST147-KL64, with only 14 SNPs (median; range, 10 to 26) (Fig. S2). This indicated that ST11-KL64 clade I acquired the large region from ST147-KL64 by recombination. Notably, five non-ST147-KL64 strains, comprising one each of ST147-KL106, ST147-KL174, and ST2358-KL64 and two of ST11-KL107, were also clustered with ST11-KL64 clade I and ST147-KL64 (Fig. S6). However, the confidence level of the capsular typing for the two ST11-KL107 strains was zero, and therefore the two strains were excluded from analysis to avoid the generation of misleading information (Fig. S6). For ST147-KL106, ST147-KL174, and ST2358-KL64, only one to three strains were available, which did not allow us to perform robust dating analysis. Nonetheless, the earliest documented recovery dates of ST147-KL106, ST147-KL174, and ST2358-KL64 strains were later than the predicted emergence of ST11-KL64 clade I (Table S1), and therefore the three types were unlikely to be the donor of the 483-kb region for ST11-KL64 clade I. Collectively, ST11-KL64 clade I is likely a hybrid, with 91.2% (ca. 4.98 Mb) of the chromosome derived from ST11-KL15 and 8.8% (483 kb) acquired from ST147-KL64 (a scheme is shown in Fig. 5). The acquisition of the 483-kb region caused a capsule switch from the KL15 to the KL64 type.

The 483-kb region contains 418 coding sequences (CDs) encoding capsule polysaccharide (CPS), lipopolysaccharide (LPS), MdtABCD multidrug efflux pump, DNA damage-repairing system RuvABC, type IV secretion system, two-component systems BaeSR and BtsSR (formerly YehUT), histidine biosynthesis, and multiple systems for transporting or acquiring various substances, such as amino acids (e.g., arginine and tyrosine), l-arabinose, iron, oligopeptides, and zinc (Data Set S8).

### ST11-KL64 clade II emerged from ST11-KL47 by acquiring a 157-kb region containing the capsule gene cluster from CC1764-KL64.

We also investigated the origin of ST11-KL64 clade II using the same approach for clade I as described above. The phylogenomic analysis identified ST11-KL47 as the sister lineage of ST11-KL64 clade II (Fig. 2). The MRCA of ST11-KL47 was dated to September 1917 (95% CI, October 1898 to September 1935), much earlier than the emergence of ST11-KL64 clade II (Fig. S7). Notably, as demonstrated in Fig. S7, a ST11-KL47 strain (accession number GCA_015022235.2) collected from Taiwan in 2014 was an outstanding outgroup separated by a long branch and had >600 SNPs from all other ST11-KL47 strains. Once this strain was excluded, the MRCA of all remaining ST11-KL47 strains was dated to June 1999 (95% CI, June 1996 to April 2002), which is still earlier than the emergence of ST11-KL64 clade II (Fig. S8).

Among the 565 genomes belonging to the branch containing all ST11-KL64 (Fig. S1), there were 1,007 SNPs (median; range, 984 to 1,171) between ST11-KL64 clade II and ST11-KL47 strains (*n* = 55). Most (*n* = 934) of these SNPs concentrated in a capsular gene cluster containing the 157-kb chromosomal region, which accounted for 3% of the complete chromosome, corresponding to nucleotide positions 1,696,593 to 1,853,327 of the chromosome of reference strain 090357 (accession number CP066523) (Fig. S9). This suggested that the 157-kb region of ST11-KL64 clade II was acquired from another origin instead of ST11-KL47, which was likely due to recombination.

**FIG 6:**
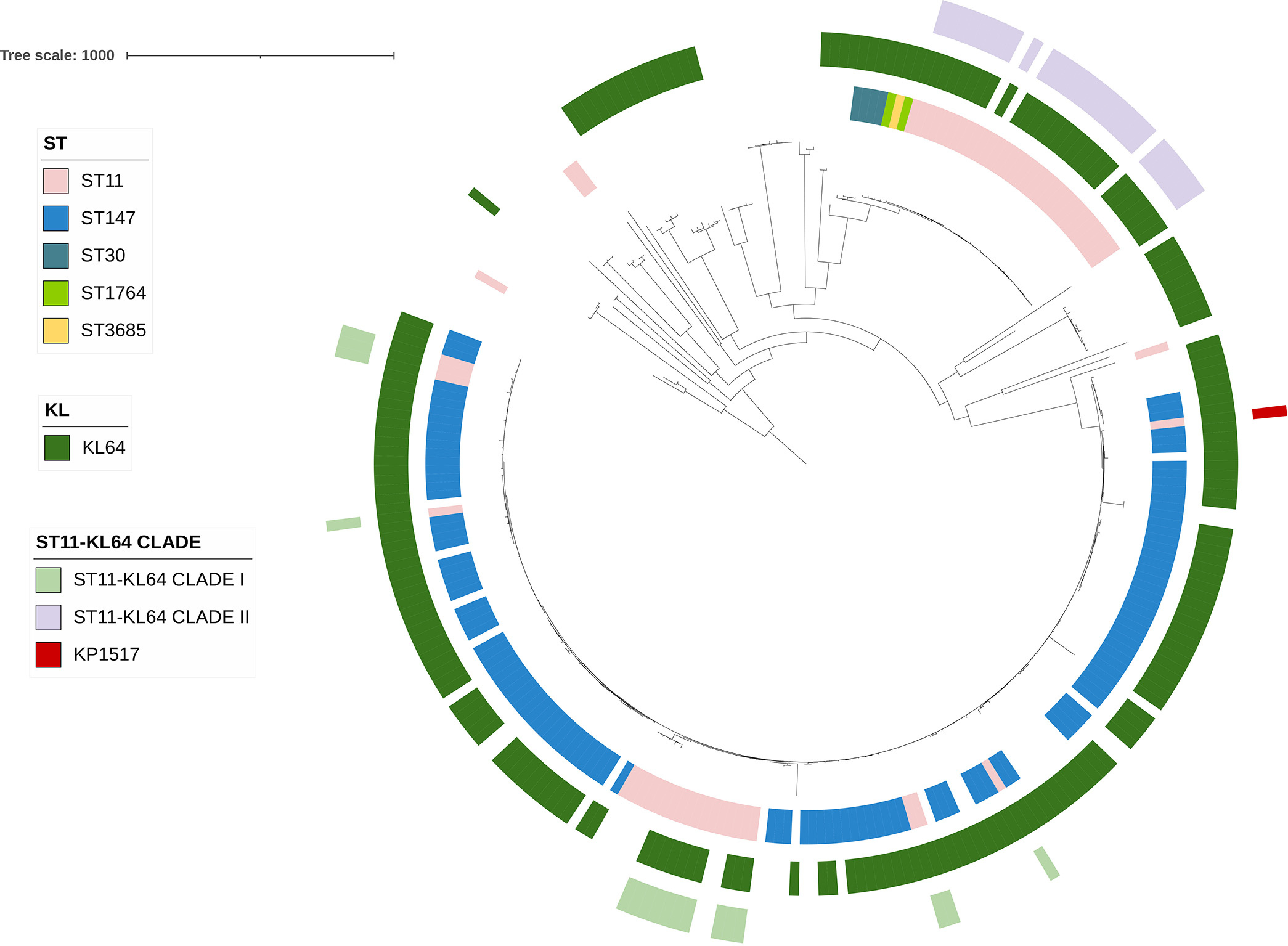
The phylogenetic tree of 301 K. pneumoniae strains based on the 157-kb recombination region. The tree was inferred using strain 090357 (accession number CP066523) as the reference. The phylogeny was inferred from core SNPs under a GTR model with site rate variation and a 100-bootstrap test. The tree was midpoint-rooted with bootstrap support of over 50% shown in gradients. The circles from the outer to the inner represent ST11-KL64 clades, KL64 strains (regardless of STs), and ST11, ST30, or ST147, respectively. The scale bar represents the number of nucleotide substitutions per site.

We therefore performed a phylogenetic analysis for the 157-kb region of the 4,826 more-unique K. pneumoniae genomes to identify the possible donor for recombination. The first iteration of Gubbins identified 301 genomes belonging to the branch containing all ST11-KL64 ones in the phylogenetic tree based on the alignment of this 157-kb region (Fig. S10). The 301 genomes were further selected for completing all five iterations of Gubbins to infer a fine-scale phylogenetic tree (Fig. 6). ST30-KL64 (four genomes), ST1764-KL64 (two), ST3685-KL64 (one), and ST11-KL107 (three) were identified as the sister lineages of ST11-KL64 clade II. The three ST11-KL107 were excluded from analysis since the confidence level of the capsular typing for the two ST11-KL107 strains was “none” (Fig. S11). The 157-kb region was highly similar between ST11-KL64 clade II and ST30-KL64 (with 4 to 40 SNPs; median of 31), ST1764-KL64 (5 SNPs), and ST3685-KL64 (4 SNPs). Notably, all of ST30, ST1764, and ST3685 belonged to a common clonal complex, CC1764 (13 in total). There were only one ST3685-KL64, four ST30-KL64, and six ST1764-KL64 strains available (Table S2) among the 12,586 K. pneumoniae genomes even before dereplicating, which did not allow us to perform robust dating analysis. Nonetheless, the earliest documented recovery date of CC1764-KL64, which was ST30-KL64, was 2002 (Table S3), earlier than the predicted emergence of ST11-KL64 clade II. The above findings suggested that ST11-KL64 clade II is very likely to be derived from ST11-KL47 by swapping a 157-kb region containing the capsule gene cluster with that of CC1764-KL64 by recombination (Fig. 5), causing capsule switch.

Notably, the 157-kb region is located in the aforementioned 483-kb region identified for clade I. The 157-kb region contains 128 CDs encoding CPS, LPS, MdtABCD efflux pump, BaeSR and BtsSR, histidine biosynthesis, and oligopeptide transporter, but not RuvABC, type IV secretion system, nor transporting or acquiring systems for amino acids, l-arabinose, iron, or zinc (Data set S8).

### The singleton KP1517 was derived from ST11-KL47 by acquiring a 126-kb region containing the capsule gene cluster from ST11-KL64 clade I.

We then investigated the origin of the singleton KP1517. ST11-KL47, rather than ST11-KL64 clade II, was the sister lineage of KP1517 in the phylogenomic tree (Fig. 2). This suggested that KP1517 and ST11-KL64 clade II were independently derived from ST11-KL47. Among the 565 genomes, there were 830 SNPs (median; range, 786 to 1,003) between KP1517 and ST11-KL47 strains (*n* = 55). Most (*n* = 760) of these SNPs were concentrated in a 126-kb chromosomal region, corresponding to nucleotide positions 1,737,170 to 1,863,290 of the 090357 chromosome (accession number CP066523), accounting for 2.3% of the complete chromosome. This 126-kb region also contains the capsule gene cluster (Fig. S12), and the concentration of SNPs in this region highlights a possible foreign origin acquired by recombination.

We also performed a phylogenetic analysis for the 126-kb region of the 4,826 more-unique K. pneumoniae genomes to identify the possible donor for recombination. The first iteration of Gubbins identified 264 genomes belonging to the branch containing all ST11-KL64 ones in the phylogenetic tree based on the alignment of this 126-kb region (Fig. S13). The 264 genomes were further selected for completing all five iterations of Gubbins to infer a fine-scale phylogenetic tree (Fig. S14). ST11-KL64 clade I was identified as the sister lineage of KP1517. The 126-kb region was highly similar between KP1517 and ST11-KL64 clade I, with 5 SNPs (median; range, 2 to 16). The 126-kb region was also highly similar between KP1517 and ST147-KL64 with 5 SNPs (median; range, 2 to 88), but the SNP range was broader than that between KP1517 and ST11-KL64 clade I. The above findings illustrated that KP1517 evolved from ST11-KL47 by swapping a 126-kb region containing the capsule gene cluster with ST11-KL64 clade I by recombination (Fig. 5).

The 126-kb region was also located in the aforementioned 483-kb region and overlapped the 157-kb region identified for clade II for 116 kb. The 126-kb region contains 102 CDs, 91 of which are also contained by the 157-kb region. The 102 CDs encode CPS, LPS, MdtABCD efflux pump, BaeSR, histidine biosynthesis, and an oligopeptide transporter but not BtsSR, based on comparison of CDs encoded by the 157-kb region (Data Set S8).

## DISCUSSION

ST11 is of particular importance and has been proposed as the basal lineage of the worldwide-spread clonal complex 258 comprising ST258, ST512, and a few closely related STs (13). In this study, we performed detailed genome-based analysis to address the origins of ST11-KL64, which is the major type of CRKP in China. We found that there were two highly supported distinct major clades and an additional singleton of ST11-KL64 with different origins. Clade I was mainly seen in Brazil and China (Taiwan) and was derived from ST11-KL15 but had exchanged a large 483-kb region containing the capsule gene cluster with ST147-KL64. Clade II was successful in mainland China and was derived from ST11-KL47 through replacement of a 157-kb region containing the capsule gene cluster from CC1764-KL64. The singleton was also derived from ST11-KL47, but independently from ST11-KL64 clade II, by acquiring a 126-kb region containing the capsule gene cluster from clade I. It became evident that the KL64 capsule gene cluster of the two ST11 clades and the additional singleton were acquired from different sources. The above findings also highlighted that a single K. pneumoniae lineage, ST11-KL47 in this case, could swap large regions containing the capsule gene cluster from various lineages to form different derivative clades.

During the preparation of the manuscript for our study, a similar genome-based study addressing the origin of ST11-KL64 K. pneumoniae was published (14). Although we used a different approach for analysis, our findings are largely consistent with those in that study (14), and we also provided additional insights on the origin of ST11-KL64 K. pneumoniae. In that study (14), it was also found that the vast majority of ST11-KL64 strains in China, corresponding to clade II in our study, originated from ST11-KL47 by recombination of a 154-kb region (positions 4,060,800 to 4,214,550 of strain KP47434 [accession number QURI00000000]; a 157-kb region in our study, positions 4,060,800 to 4,217,540) containing the capsule gene cluster with CC1764-KL64. Strain HB25-1, which was regarded as an infrequent subclone in that study (14) but was assigned to clade I in our study, was found to obtain a 485-kb region (positions 1,676,640 to 2,161,600 [accession number CP039524]; a 483-kb region in our study, positions 1,678,192 to 2,161,174) containing the capsule gene cluster from ST147-KL64. These two major findings were consistent between our study and that of Chen et al. (14), indicating the robustness of the findings. The slight differences between the regions identified in the two studies were present in the boundary and were likely due to the use of different approaches for identification of the recombination regions (Gubbins in our study and SNP calling by Chen et al. [14]).

Notably, the study of Chen et al. (14) focused on strains from China, while we included all available ST11-KL64 genomes regardless of the geographical locations, considering that ST11-KL64 is globally distributed (8, 9). By this approach, we were able to find that the clade comprising HB25-1 was not infrequent outside China but represented a major clade of ST11-KL64, which was more common in Brazil. We also illustrated that the clade comprising HB25-1 was not derived from ST11-KL47 but from ST11-KL15 instead. We further identified a singleton in addition to the aforementioned two major clades to provide a comprehensive view of ST11-KL64. Our additional findings highlighted the importance of the inclusion of all available genomes regardless of geographical locations to address the origins of bacterial lineages.

Chromosomal recombination events resulting in replacement of large chromosomal regions have been well documented in K. pneumoniae, an they are associated with successful clones. For instance, an ancestor of ST11 acquired a 1.3-Mb contiguous region from ST1628 by recombination, which was estimated to have occurred before 1985, formed the basal lineage of CG258 comprising ST11 and ST258 (11). ST258 K. pneumoniae is a hybrid which emerged due to the replacement of approximately 20% of the ST11 genome by a 1.1-Mb contiguous chromosomal fragment from an ST442 donor strain (11). Further homologous recombination of the capsule gene region from an ST42 strain formed another clade of ST258 (11). A previous study of a limited collection of ST11-KL64 CRKP strains (*n* = 80), all of which were from China, reported that ST11-KL64 could have emerged from ST11-KL47 in 2011 (11). However, ST11-KL64 has also been reported in a few other countries, as described above. In this study, we included all available ST11-KL64 genomes for analysis and found that ST11-KL64 was in fact a heterogenous lineage comprising two major clades and a singleton. The two clades and the singleton have different origins and emerged at different places at different time points, which may reflect the flexible adaptation of major human pathogens such as K. pneumoniae to the varied survival pressures, such as selection pressure imposed by antimicrobial use in different places.

The three (483-, 157-, and 126-kb) recombination regions contributing to the formation of the two major ST11-KL64 clades plus a singleton contain a common 110-kb region with 91 common CDs. The CDs encode CPS, LPS, MdtABCD efflux pump, BaeSR, histidine biosynthesis, and oligopeptide transporter. CPS is well known as a key factor associated with the pathogenicity of K. pneumoniae (15, 16). LPS is also considered important for Klebsiella pathogenicity, in particular in bacteremia (16), and interacts with CPS (17). Histidine biosynthesis plays a critical role in many cellular enzymatic reactions and metabolisms (18). The two-component system BaeSR senses and responds to various stimuli (19) as well as regulates MdtABCD (20), an efflux pump that protects bacterial cells from various environmental toxins (21, 22). Oligopeptide transporters recycle cell wall peptides and play an important role in nutrition for bacteria (23). These CDs are associated with bacterial virulence, colonization, growth, and survival under various hostile conditions and may contribute to the spread of ST11-KL64 K. pneumoniae across and in health care settings. Further studies of the 110-kb region encoding these CDs are warranted and may uncover mechanisms driving the spread and dominance of ST11-KL64 K. pneumoniae.

We are aware of limitations in this study. First, we used publicly available genomes, which are largely biased and commonly not well-curated. The dating estimation can be significantly impacted by the available metadata and therefore may not be correct. Second, we used assemblies rather than raw reads for the phylogenomic dating analysis with Snippy. Snippy utilized wgsim-converted pseudo-reads for SNP analysis, which may have introduced uncertainty if the quality of the assembly was poor (https://github.com/tseemann/snippy). Third, we did not find the exact mechanisms for the recombination events.

Chromosomal regions containing the capsule gene cluster appear to be a hot spot of recombination in K. pneumoniae (5). We identified new recombination events contributing to the understanding of CRKP evolution, providing novel insights into the emergence of high-risk clones. Our findings highlight that recombination between such regions represents a major evolutive mechanism employed by K. pneumoniae for rapid evolution to lead to novel clades to accommodate stress for survival by altering antigenic presentation and ultimately diverting the host response (6, 24). Some of these clades become particularly successful, like ST11-KL64 clade II in China. After understanding the origins, further studies are warranted to closely monitor the evolution of high-risk clades to inform the design of targeted countermeasures.

## MATERIALS AND METHODS

### Global data set of publicly available genome sequences.

All publicly available K. pneumoniae genome assemblies in NCBI as of 1 June 2022 (*n* = 13,625) were retrieved and were assessed for completeness and heterogeneity using CheckM v1.1.10 (25) (Data Set S1). Genomes with G+C content and genome size not matching K. pneumoniae, with 95% completeness, >50% heterogeneity, or >5% contamination were discarded from further analysis.

### Inferring recombination-free phylogenomic trees.

After quality control, all included K. pneumoniae genomes (*n* = 12,586) (Data Set S2) were subjected to dereplicating genome assemblies using dereplicator.py (https://github.com/rrwick/Assembly-Dereplicator), a Python script for removing redundant assemblies that are sufficiently closely related as defined by a distance threshold. The generated more-unique genomes (*n* = 4,826) were assigned to sequence types and capsular types using Kleborate v2.2.0 (26). After removing recombination regions using Gubbins v3.1.6 (27), these genomes (Data Set S3) were included for phylogenomic analysis using rapidnj (https://birc.au.dk/Software/RapidNJ/) and the reconstruction of ancestral sequences was performed using RAxML-NG (https://github.com/amkozlov/raxml-ng). Based on the phylogenomic tree inferred by the first iteration of Gubbins (Fig. S1), genomes (*n* = 565) belonging to the branch containing all ST11-KL64 ones were further selected for completing all five iterations of Gubbins to infer a fine-scale phylogenomic tree (Fig. 1). The tree was tested using 100 bootstraps in RAxML v8.2.12 (28) under the GTR model and was visualized and annotated using iTOL v3 (29) and Phandango v1.3.0 (30). Genes encoding known carbapenemases were predicted using Kleborate v2.2.0 (26).

### SNP calling and analysis of the recombination regions.

A ST11-KL64 CRKP strain, 090357, with a complete chromosome sequence (accession number CP066523) reported by us previously (31), was used as a reference for calling core genome SNPs by Snippy v4.6.0 (https://github.com/tseemann/snippy). The distribution of SNPs and the putative recombination region were visualized using Harvest (32) and Gubbins, respectively. To identify potential donor strains of the recombination, we retrieved sequences of the recombination region from aforementioned 4,826 genomes generated after dereplicating genome assemblies using dereplicator.py for alignment using Snippy. A phylogenetic tree based on the alignment of this region was inferred by the first iteration of Gubbins and the genomes belonging to the branch containing all ST11-KL64 ones, which were further selected for completing all five iterations of Gubbins to infer a fine-scale phylogenetic tree. The tree was tested using 100 bootstraps with a GTR model (33) and was visualized and annotated using iTOL v3 (29) and Phandango v1.3.0 (30).

### Coalescent analysis for dating the emergence of ST11-KL64.

All of the ST11-KL64, ST11-KL47, ST11-15, and ST147-KL64 strains in the 12,586 included K. pneumoniae genomes were selected for the coalescent analysis. These strains were fed into Gubbins (27) to obtain a recombination-corrected phylogenomic tree, which was then input into BactDating v1.0.1 (12) under a mixedcarc model (27) with 10^7^ iterations to ensure that the effective sample size of the inferred parameters α, μ, and σ were >200 in the run of the Markov chain Monte Carlo analysis.
