# Supplementary material for: The Origins of ST11 KL64 Klebsiella pneumoniae: a Genome-Based Study
Source: Microbiol Spectr. 2023 Mar 27;11(2):e04165-22. doi: 10.1128/spectrum.04165-22 (PMC10101065; doi:10.1128/spectrum.04165-22)

Supplementary files

Table S1. The collection date of ST147-KL106 and ST2358-KL64 strains.
Table S2. The collection date of ST30-KL64 strains.
Table S3. The collection date of ST1764-KL64 and ST3685-KL64 strains.

Fig S1. The first iteration phylogeny tree of the 4,826 more unique *K. pneumoniae* genomes.
Fig S2. Phylogenetic tree and the distribution of SNPs among ST11-KL64 clade I, ST11-KL15, and ST147-KL64 within the 4,826 more unique *K. pneumoniae* genomes.
Fig S3. The dated phylogenomic tree of the 105 ST11-KL15 strains.
Fig S4. The first iteration phylogeny tree of the 4,826 more unique *K. pneumoniae* genomes based on the 482-kb recombination region.
Fig S5. The dated phylogenomic tree of 375 ST147-KL64 strains.
Fig S6. Five non-ST147-KL64 strains clustering with ST11-KL64 clade I and ST147-KL64 in the phylogenetic tree of 857 *K. pneumoniae* strains based on the 482-kb recombination region.
Fig S7. The dated phylogenomic tree of 409 ST11-KL47 strains.
Fig S8. The dated phylogenomic tree of 408 ST11-KL47 strains with exclusion of an outgroup.
Fig S9. Phylogenetic tree and the distribution of SNPs among ST11-KL64 clade II, ST11-KL47 and ST30-KL64 within the 4,826 more unique *K. pneumoniae* genomes.
Fig S10. The first iteration phylogeny tree of the 4,826 more unique *K. pneumoniae* genomes based on the 157-kb recombination region.
Fig S11. Six non-ST30-KL64 strains clustering with ST11-KL64 clade II and ST30-KL64 in the phylogenetic tree of 301 *K. pneumoniae* strains based on the 157-kb recombination region.
Fig S12. Phylogenetic tree and the distribution of SNPs among KP1517, ST11-KL64 clade I, and ST11-KL47 within the 4,826 more unique *K. pneumoniae* genomes.
Fig S13. The phylogenetic tree of the 4,826 more unique *K. pneumoniae* genomes based on the 126-kb recombination region.
Fig. S14. The phylogenetic tree of 264 *K. pneumoniae* strains based on the 126-kb recombination region.

Dataset S1. The original 13,625 *K. pneumoniae* genome assemblies in NCBI as of June 1, 2022.
Dataset S2. The 12,586 *K. pneumoniae* genomes after quality control.
Dataset S3. The 730 ST11-KL64 *K. pneumoniae* genomes.
Dataset S4. The 4,826 more unique *K. pneumoniae* genomes after dereplicating.
Dataset S5. The 43 ST11-KL64 genomes within the 4,826 more unique *K.*

pneumoniae genomes after dereplicating.
Dataset S6. The 565 genomes belonging to the branch containing all ST11-KL64 ones in the phylogenomic tree inferred by the first iteration of Gubbins.
Dataset S7. Sequence types of KL64 strains.
Dataset S8. Coding sequences in the 483-, 157-, and 126-kb recombination regions.

Table S1. The collection date of ST147-KL106 and ST2358-KL64 strains.

**Accession no.**[a]	**ST**	**KL**	**K_locus_confidence**	collection date
GCA_005503935.1	ST147	KL106	Very high	2017
GCA_900511135.1	ST147	KL106	Good	2014
GCA_911728565.1	ST147	KL106	Good	2016
GCA_900493375.1	ST147	KL174	Good	2016
GCA_022501275.1	ST2358	KL64	Perfect	2019/Jan
GCA_022501265.1	ST2358	KL64	Perfect	2019/Jan

[a]The three genomes clustered with ST11-KL64 clade I in Fig. 4 are underlined.

Table S2. The collection date of ST30-KL64 strains.

**Accession no.**	**ST**	**KL**	**K_locus_confidence**	collection date
GCA_902507545.1	ST30	KL64	Very high	2017
GCA_002853795.1	ST30	KL64	Very high	2002
GCA_900500975.1	ST30	KL64	Very high	2013
GCA_904863125.1	ST30	KL64	Very high	/

Table S3. The collection date of ST1764-KL64 and ST3685-KL64 strains.

**Accession no.**[a]	**ST**	**KL**	**K_locus_confidence**	**Collection Date**
GCA_013283935.1	ST1764	KL64	Very high	2019/May
GCA_902507285.1	ST1764	KL64	Very high	2012
GCA_902508495.1	ST1764	KL64	Very high	2017
GCA_021648805.1	ST1764	KL64	Very high	2016
GCA_902509385.1	ST1764	KL64	Very high	2017
GCA_019401225.1	ST1764	KL64	Very high	2015/Dec
GCA_004120155.1	ST3685	KL64	Very high	2014/Sep

[a]The three genomes clustered with ST11-KL64 clade II in Fig. 6 are underlined.

Fig S1. The first iteration phylogeny tree of the 4,826 more unique *K. pneumoniae* genomes. The tree was inferred using strain 090357 (accession no. CP066523) as the reference. Genomes (n=565) belonging to the branch containing all ST11-KL64 ones are shown in the green region. Scale bar represents number of nucleotide substitutions per site.

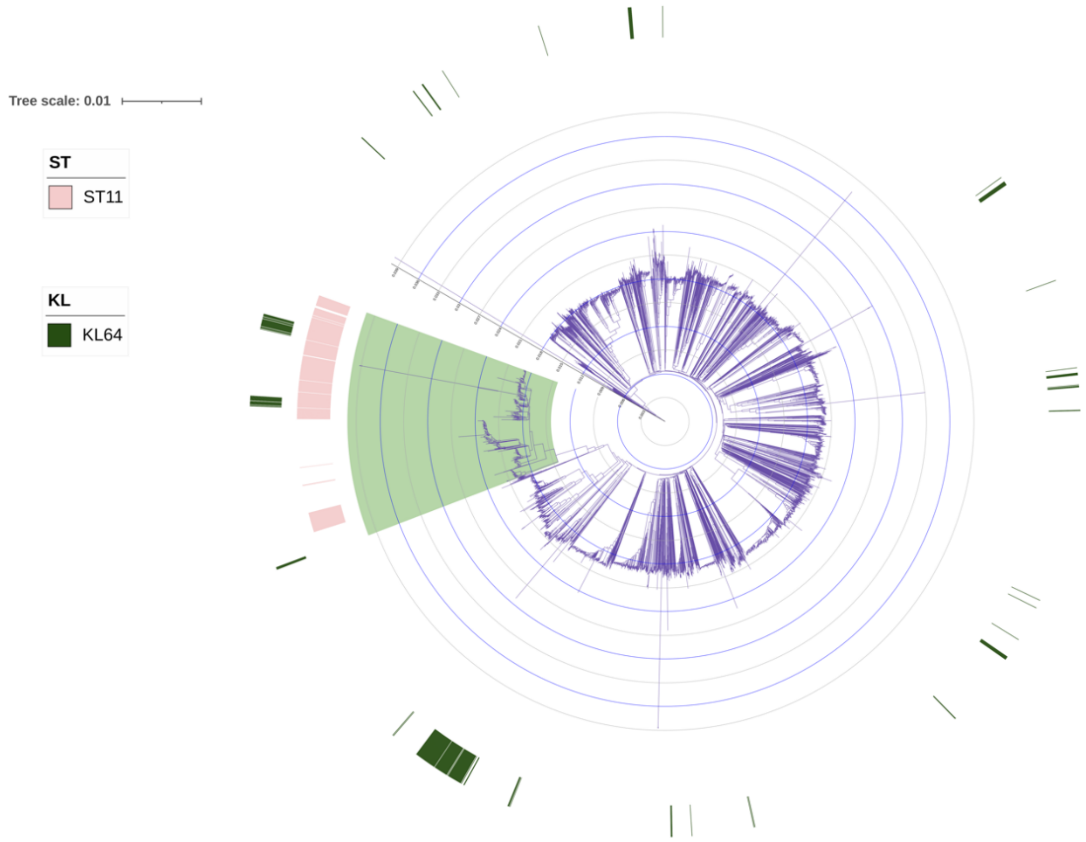

Fig S2. Phylogenetic tree and the distribution of SNPs among ST11-KL64 clade I, ST11-KL15, and ST147-KL64 within the 4,826 more unique *K. pneumoniae* genomes. Pink lines indicate SNPs identified using the harvest suite. The region of divergence is highlighted by a green box.

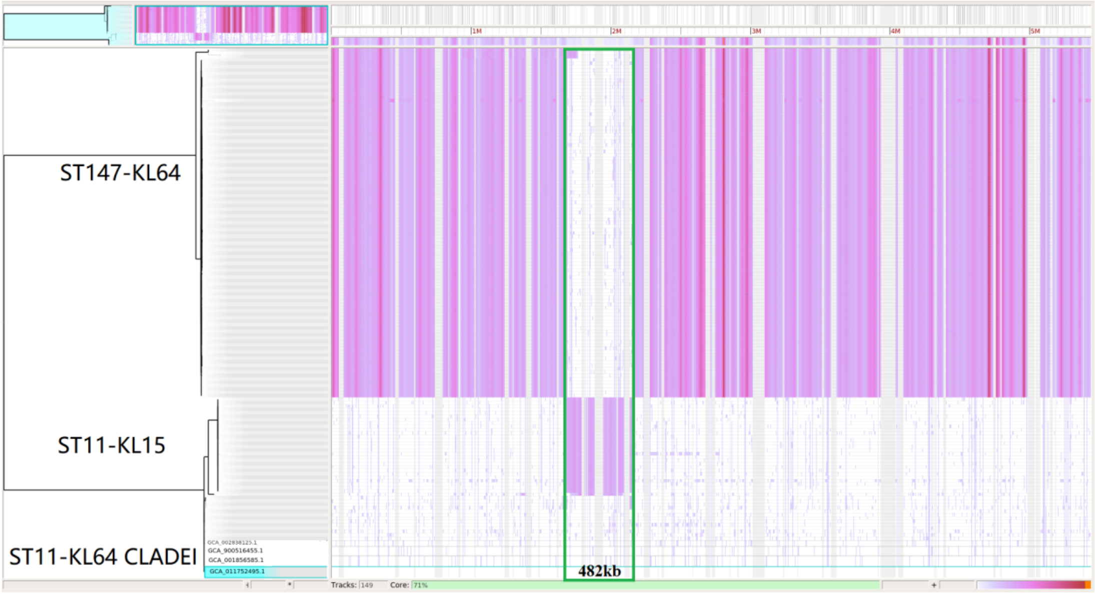

Fig S3. The dated phylogenomic tree of the 105 ST11-KL15 strains. The tree was inferred using BactDating v1.0.1 and corrected for recombination using Gubbins v3.1.6. The refined analysis revealed an average point mutation rate of μ= 5.94 (95% confidence interval [CI], 4.49–8.07) substitutions per year for ST11-KL15 strains and a root date of February 1984 (95% CI, May 1973–July 1992), which is indicated by point A here.

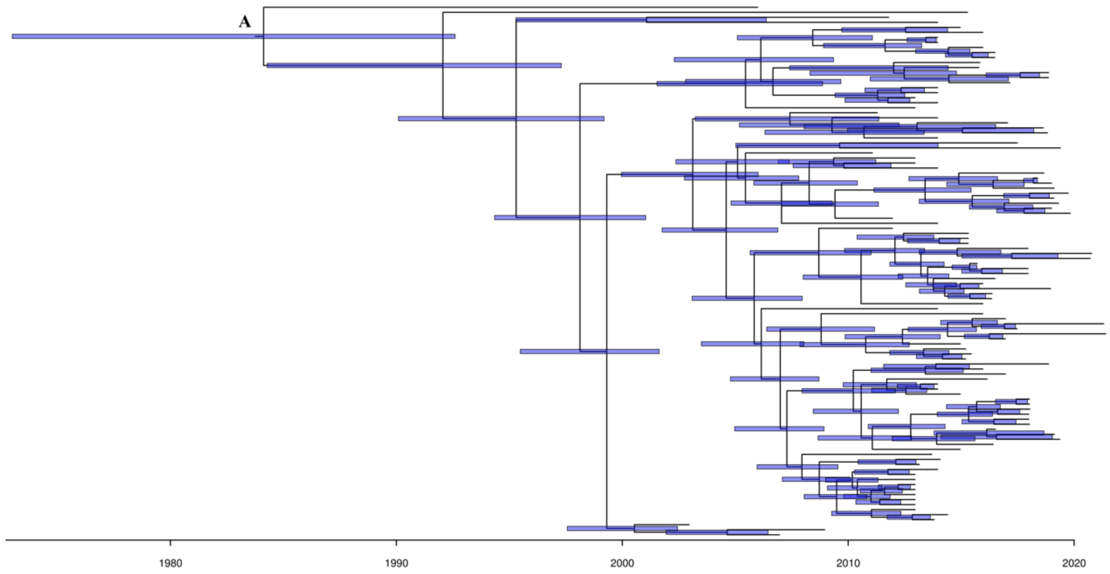

Fig S4. The first iteration phylogeny tree of the 4,826 more unique *K. pneumoniae* genomes based on the 482-kb recombination region. The tree was inferred using strain 090357 (accession no. CP066523) as the reference. Genomes (n=857) belonging to the branch containing all ST11-KL64 ones are highlighted in a green region. Scale bar represents number of nucleotide substitutions per site.

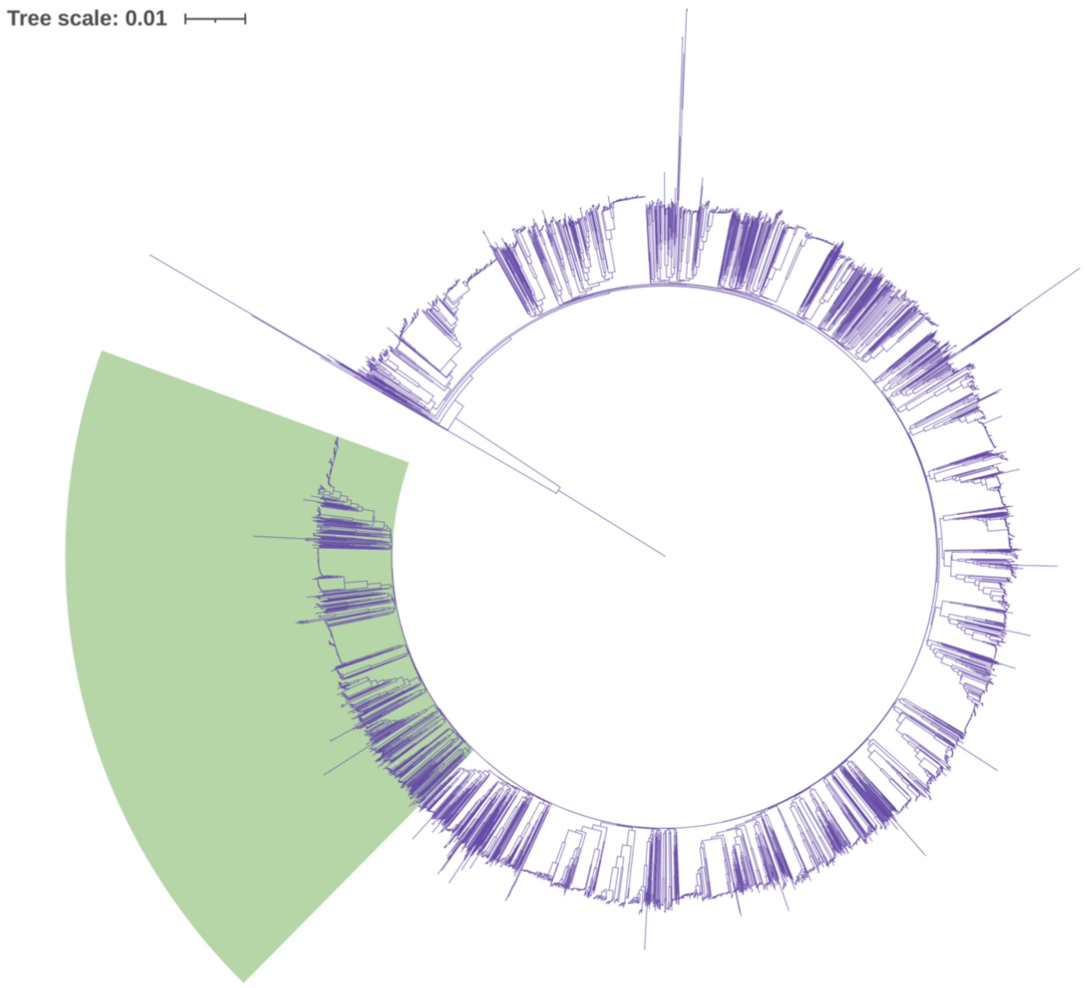

Fig S5. The dated phylogenomic tree of 375 ST147-KL64 strains. The tree was inferred using BactDating v1.0.1 and corrected for recombination using Gubbins v3.1.6. The refined analysis revealed an average point mutation rate of μ= 7.85 (95% confidence interval [CI], 6.69–8.89) substitutions per year for ST147-KL64 strains and a root date of May 1894 (95% CI, November 1866–July 1921), which is indicated by point A here.

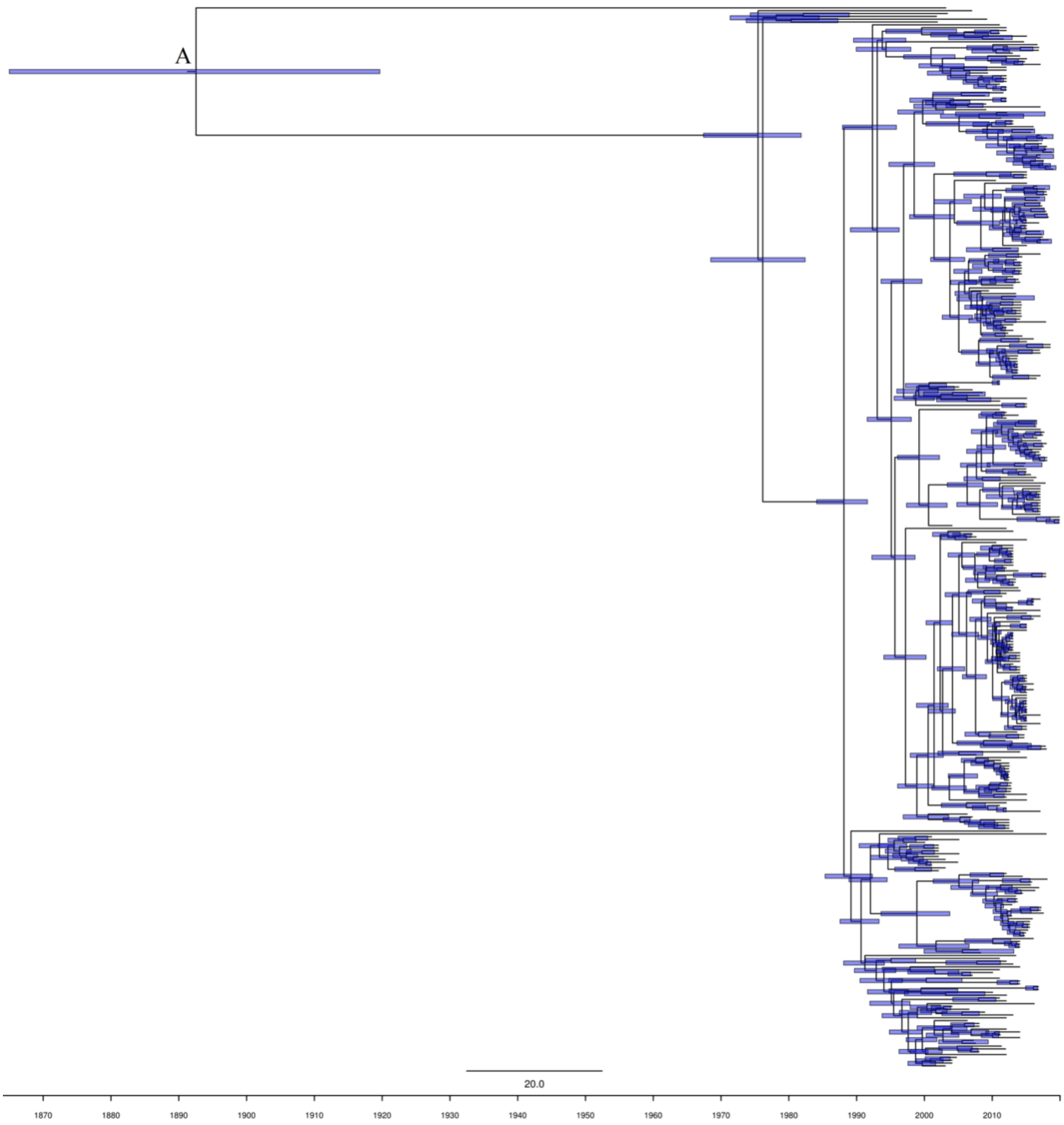

Fig S6. Five non-ST147-KL64 strains clustering with ST11-KL64 clade I and ST147-KL64 in the phylogenetic tree of 857 *K. pneumoniae* strains based on the 482-kb recombination region. Upper left panel is Fig 4 in the main manuscript and upper right panel is an enlarged view of the region containing ST11-KL64 clade I. Number 1 to 5 represents each of the five strains with the information listed below.

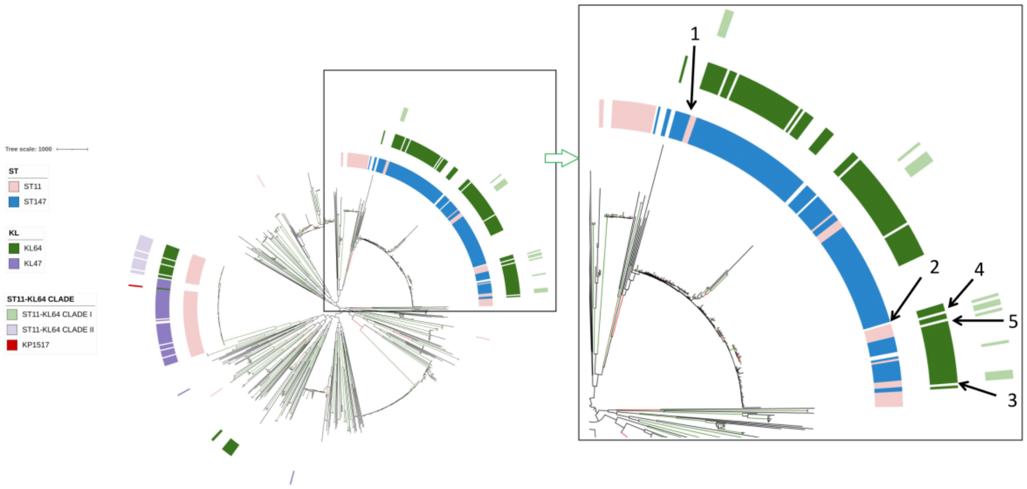

	Accession no.	ST	KL	K_locus_confidence	collection date
1	GCA_005503935.1	ST147	KL106	Very high	2017
2	GCA_022501265.1	ST2358	KL64	Perfect	2019
3	GCA_900493375.1	ST147	KL174	Good	2016
4	GCA_023061095.1	ST11	KL107	None	2020
5	GCA_003321315.1	ST11	KL107	None	2013

Fig S7. The dated phylogenomic tree of 409 ST11-KL47 strains. The tree was inferred using BactDating v1.0.1 and corrected for recombination using Gubbins v3.1.6. The refined analysis revealed an average point mutation rate of μ= 5.06 (95% confidence interval [CI], 4.48–5.65) substitutions per year for ST11-KL47 strains and a root date of September 1917 (95% CI, October 1898–September 1935), which is indicated by point A here.

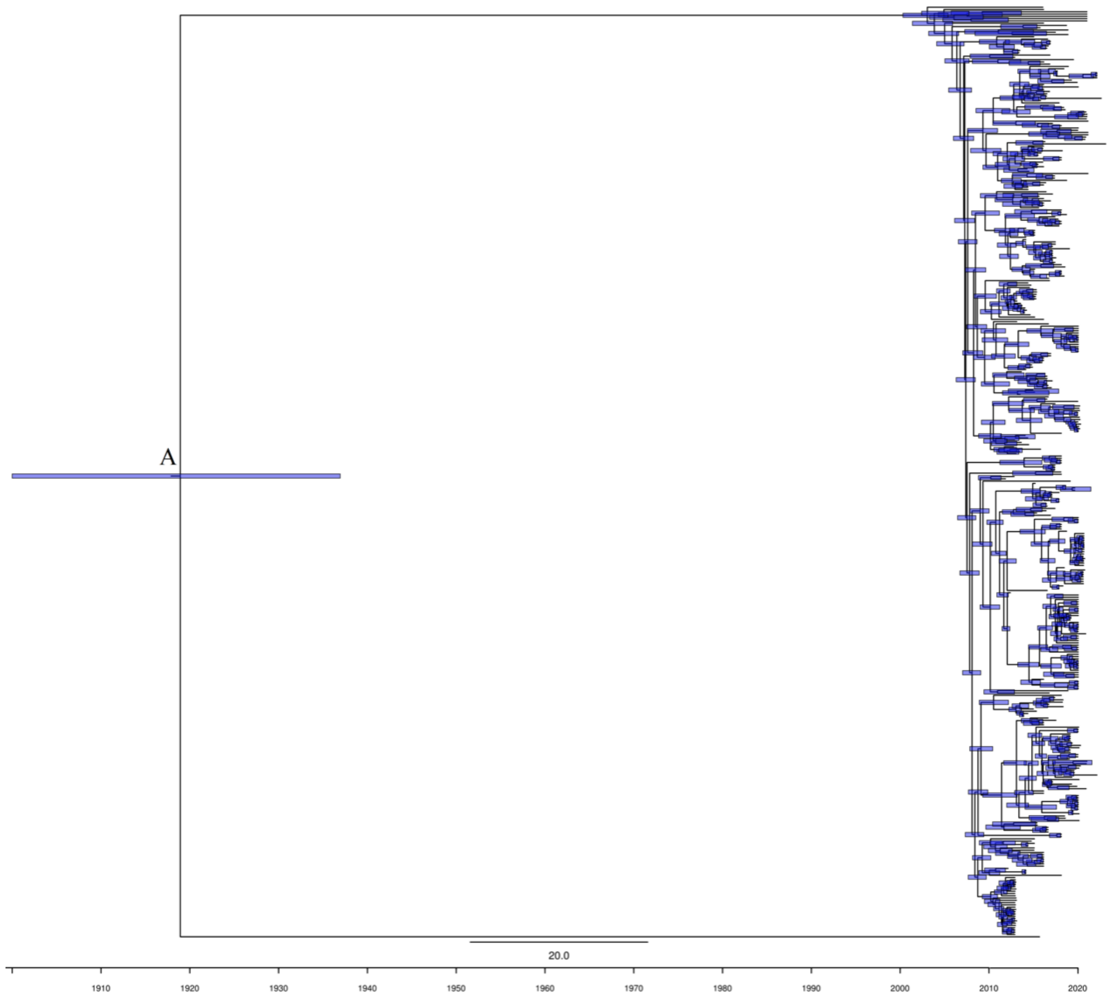

Fig S8. The dated phylogenomic tree of 408 ST11-KL47 strains with exclusion of an outgroup. The tree was inferred using BactDating v1.0.1 and corrected for recombination using Gubbins v3.1.6. The refined analysis revealed an average point mutation rate of μ= 6.04 (95% confidence interval [CI], 5.40–6.70) substitutions per year for ST11-KL47 strains and a root date of June 1999 (95% CI, June 1996–April 2002).

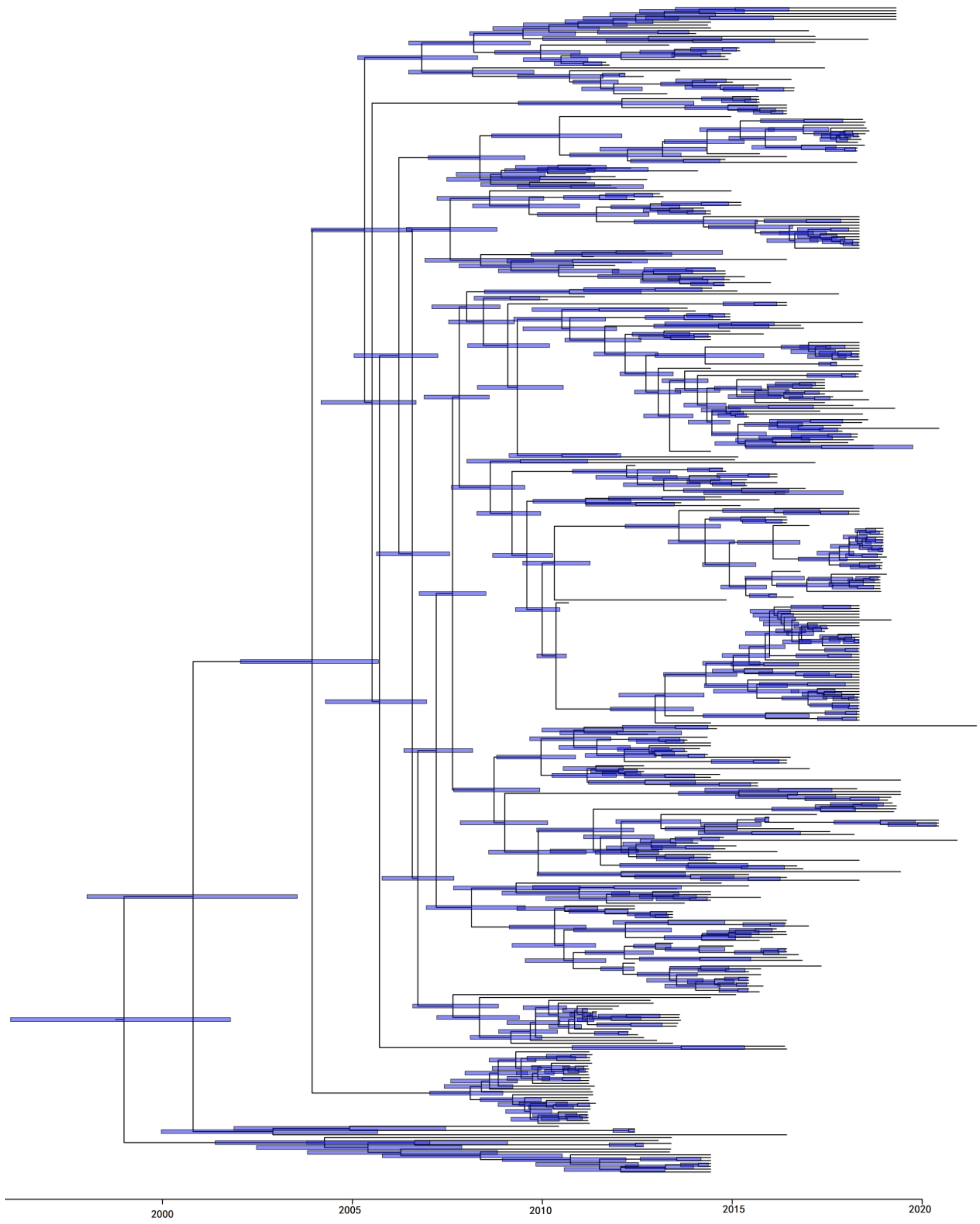

Fig S9. Phylogenetic tree and the distribution of SNPs among ST11-KL64 clade II, ST11-KL47 and CC1764-KL64 within the 4,826 more unique *K. pneumoniae* genomes. Pink lines indicate SNPs identified with the harvest suite. The region of divergence is highlighted by a green box. ST11-KL47 is highlighted by a pink box.

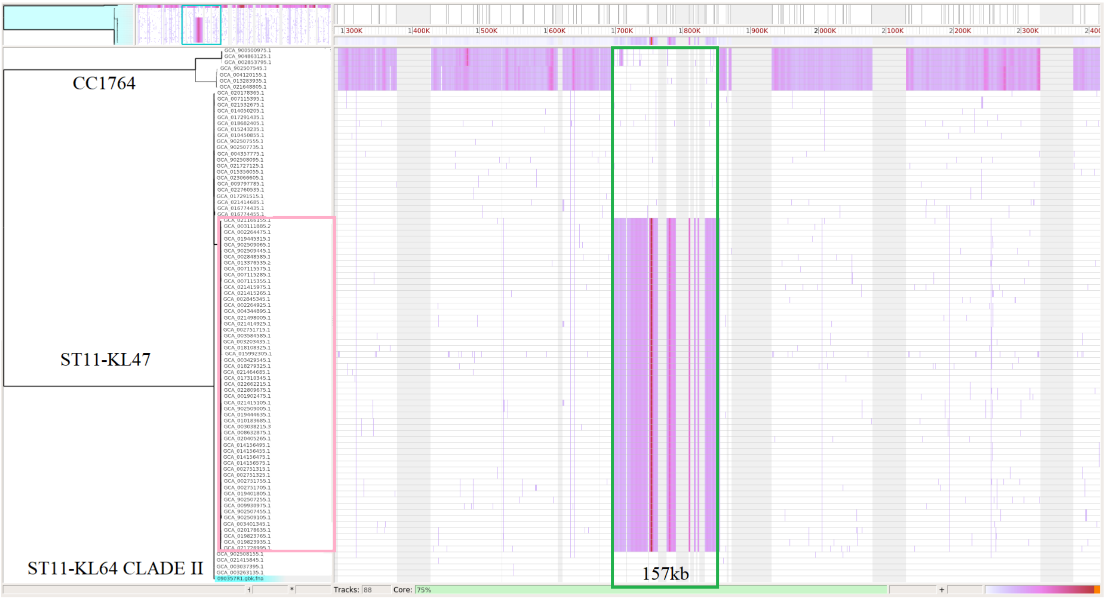

Fig S10. The first iteration phylogeny tree of the 4,826 more unique *K. pneumoniae* genomes based on the 157-kb recombination region. The tree was inferred using strain 090357 (accession no. CP066523) as the reference. Genomes (n=301) belonging to the branch containing all ST11-KL64 ones are highlighted in a green region.

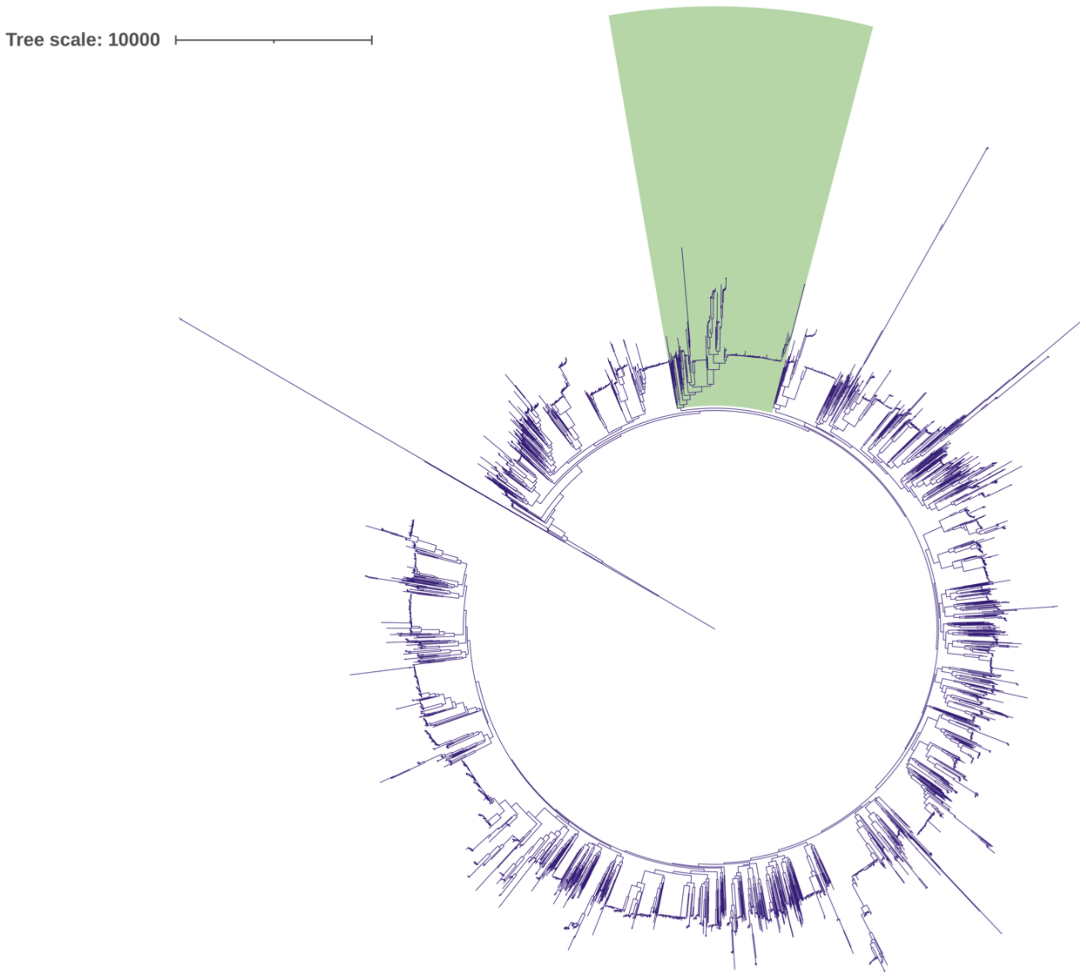

Fig S11. Six non-ST30-KL64 strains clustering with ST11-KL64 clade II and ST30-KL64 in the phylogenetic tree of 301 *K. pneumoniae* strains based on the 157-kb recombination region. Upper left panel is Fig 6 in the main manuscript and upper right panel is an enlarged view of the region containing ST11-KL64 clade II. Number 1 to 4 represents the six strains with the information listed below.

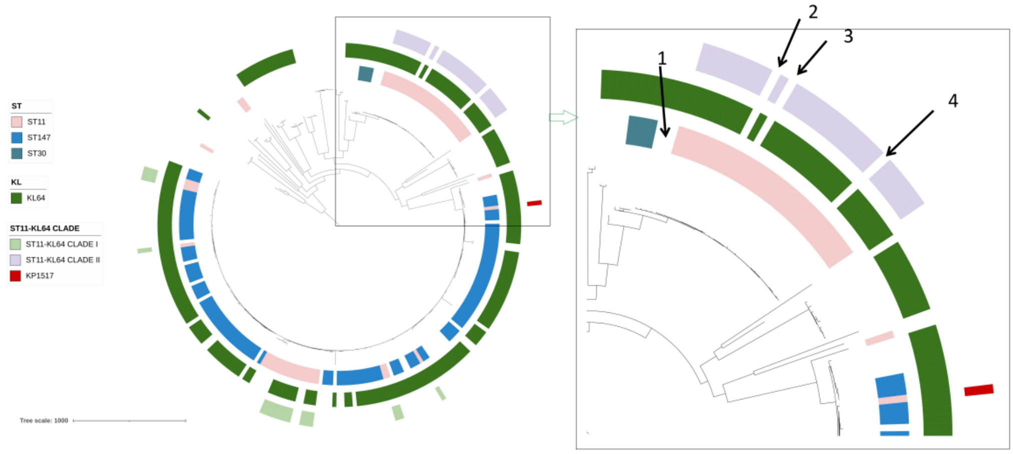

	Accession no.	ST	KL	K_locus_confidence	Collection Date
1	GCA_021648805.1	ST1764	KL64	Very high	2016
1	GCA_013283935.1	ST1764	KL64	Very high	2019
1	GCA_004120155.1	ST3685	KL64	Very high	2014
2	GCA_008375045.1	ST11	KL107	None	2018
3	GCA_003312205.1	ST11	KL107	None	2017
4	GCA_008374385.1	ST11	KL107	None	2018

Fig S12. Phylogenetic tree and the distribution of SNPs among KP1517, ST11-KL64 clade I, and ST11-KL47 within the 4,826 more unique *K. pneumoniae* genomes. Pink lines indicate SNPs identified with the harvest suite. The region of divergence is highlighted by a green box.

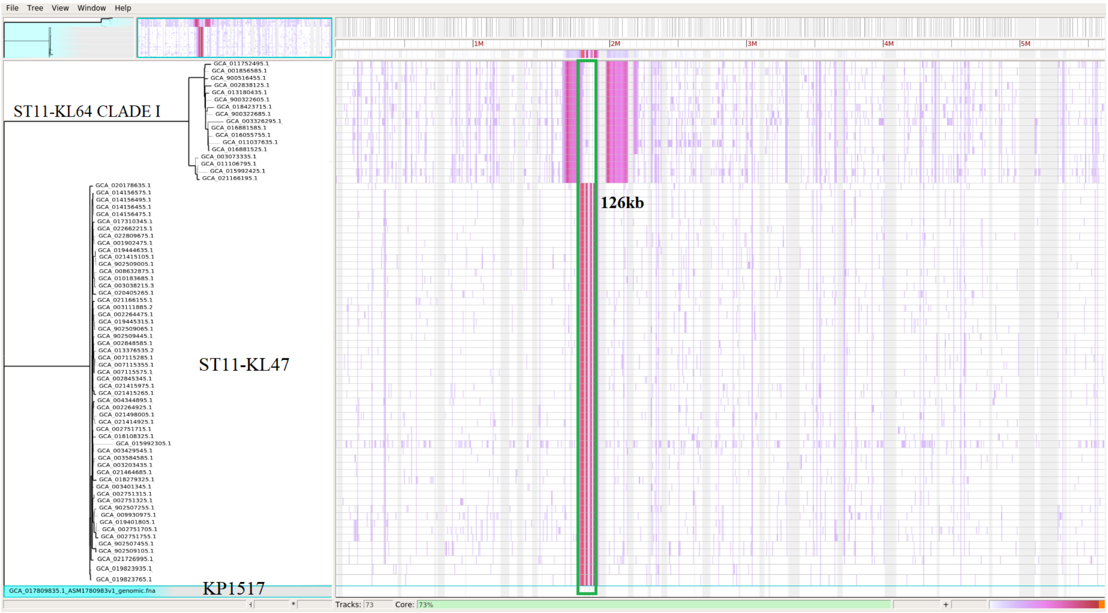

Fig S13. The phylogenetic tree of the 4,826 more unique *K. pneumoniae* genomes based on the 126-kb recombination region. The tree was inferred using strain 090357 (accession no. CP066523) as the reference. The phylogeny was inferred from core SNPs under GTR model with site rate variation and a 100-bootstrap test. The tree was midpoint-rooted with bootstrap support over 50% shown in gradients. The circles from the outer to the inner represent ST11-KL64 clades, KL64 strains (regardless of STs), and ST11 or ST147 strains, respectively. Scale bar represents number of nucleotide substitutions per site.

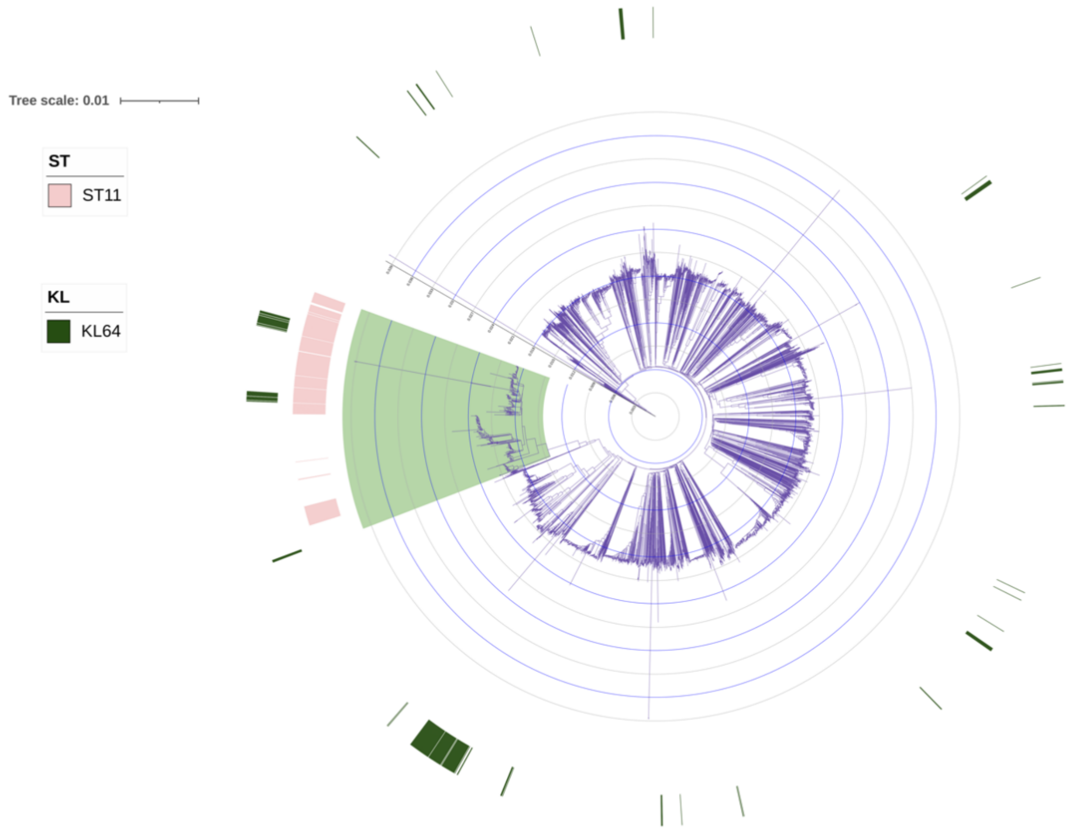

Fig S14. The phylogenetic tree of 264 *K. pneumoniae* strains based on the 126-kb recombination region. The tree was inferred using strain 090357 (accession no. CP066523) as the reference. The phylogeny was inferred from core SNPs under GTR model with site rate variation and a 100-bootstrap test. The tree was midpoint-rooted with bootstrap support over 50% shown in gradients. Scale bar represents number of nucleotide substitutions per site.

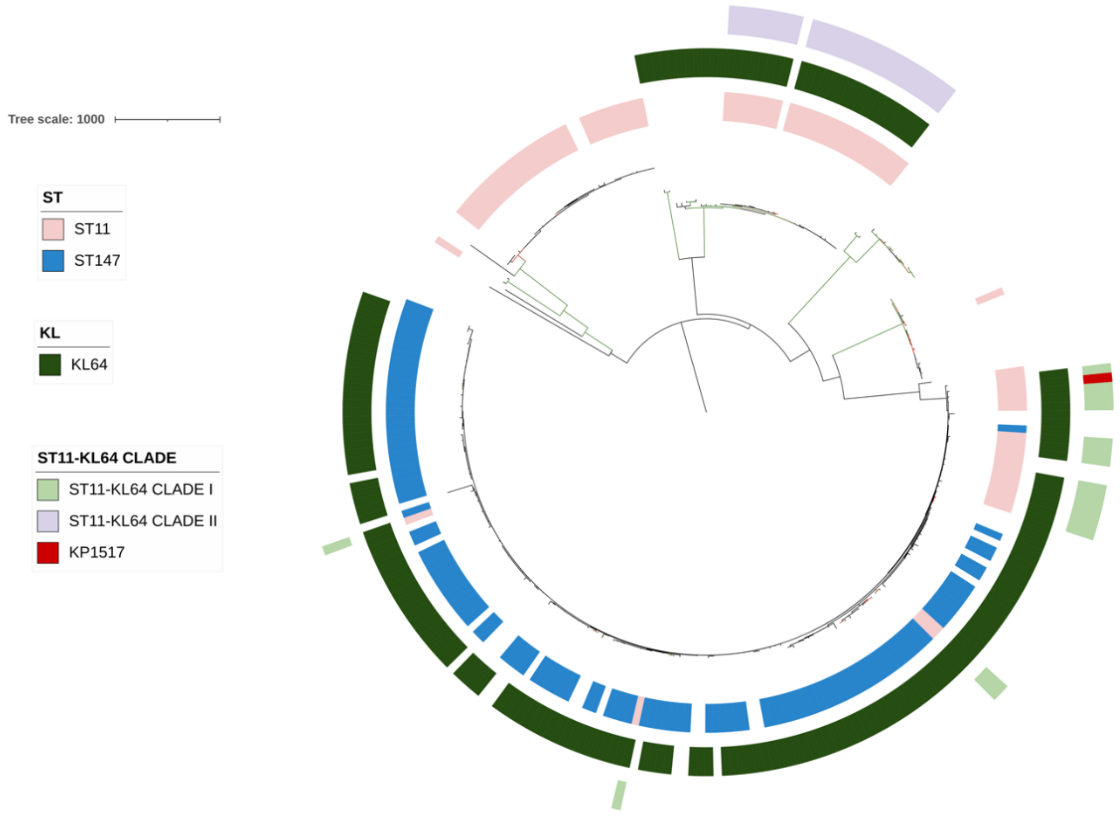

Supplement: Supplemental file 9 — Tables S1 to S3 and Fig. S1 to S14. Download spectrum.04165-22-s0001.pdf, PDF file, 3.7 MB [file spectrum.04165-22-s0001.pdf]
